# fMRI data for creativity reconfigure new conceptual knowledge through hippocampus-middle temporal gyrus

**DOI:** 10.1016/j.dib.2020.105538

**Published:** 2020-04-18

**Authors:** Jingyuan Ren, Ying Zhou, Jing Luo

**Affiliations:** a Beijing Key Laboratory of Learning and Cognition, School of Psychology, Capital Normal University, Beijing 100048, China; b Key Laboratory of Mental Health, Institute of Psychology, Chinese Academy of Sciences, Beijing 100101, China

**Keywords:** Creativity, Hippocampus, Middle temporal gyrus, Conceptual, Novelty, Usefulness, fMRI

## Abstract

Creativity is critical for human development and social progress. There is a growing interest in studies on the neural mechanism of creativity with functional magnetic resonance imaging (fMRI). However, it is hard to investigate the neural basis of creativity as high-level cognitive processing in the human brain. Based on the two-fundamental feature of novelty and usefulness in creativity [1], [2], [3], [4], [5]. We could reveal the neural mechanism of creativity to investigate how the novelty and usefulness processing during creativity activity. The dataset contains two-part of data. First, online scanning data includes the fMRI scans and T1-weighted anatomical scans acquired under participants comprehend the three type of creative designs. The creative design includes familiar and useful design (FU), novel and useless design (NS), novel and useful design (NU). Participants were asked to comprehend each design throughout the entire 6 s and respond to a usefulness evaluation for every design via pressing the yes or no button. The three types of designs were pseudo-random presented during scanning. Second, post-test data includes the behavioral data of novelty and usefulness evaluation in a 5-scaled test using the same creative design pictures in fMRI scanning by the same group of participants. The dataset is meant to be used to assess the neural basis of novelty and usefulness features processing in creativity; it also allows for empirical investigation of how the neural bases responses to the different novel signal (e.g., usefulness signal and useless signal), the human brain distinguishes the familiar or novel signal. The dataset is a supplement to the research findings in the “The function of the hippocampus and middle temporal gyrus in forming new associations and concepts during the processing of novelty and usefulness features in creative designs” published in NeuroImage [6].

Specifications Table

**Table:** 

Subject	Neuroscience
Specific subject area	Neuroimaging, fMRI, Creativity
Type of data	Tables Image Figures
How data were acquired	The imaging data T2 were acquired on a 3T Philips Achieva 3.0T TX MRI scanner with a 32-channel head coil using an echo-planar-imaging (EPI) sequence based on blood oxygenation level-dependent (BOLD) contrast. High-resolution structural T1*-weighted anatomical images of the whole-brain images were acquired using a 3D gradient-echo pulse sequence.
Data format	Raw image data include T1 and T2 and behavior data of rating novelty and usefulness scores for each scanned design are presented for each participant.
Parameters for data collection	T2*-weighted function images: repetition time (TR) = 2000 ms, echo time (TE) = 35 ms, flip angle (FA) = 90°, field of view (FOV) = 200 mm × 200 mm, 64 × 64 matrix, voxel size = 3.12 mm × 3.12 mm × 4 mm, 30 slices, 4 mm thickness, and no-gap slices. 3D -T1*-weighted anatomical images:180 slices, TR = 7.65 ms, TE = 3.73 ms, FA = 8°, FOV = 230 mm × 230 mm, voxel size = 1 mm × 1 mm × 1 mm, 1 mm thickness.
Description of data collection	Participants were asked to comprehend each design throughout the 6 s and respond to the usefulness evaluation for every design via press the yes or no button within 6 s. There were three types of creative design, familiar and useful design (FU), novel and useless design (FU), novel and useful design (NU). They performed 51 trials each for all the three conditions, and the three conditions trials are pseudo-random presentations. Each of the trials will display 6 s and disappear autumnally. Between the two trials was used jitter with 3–5 s. Also, the trials were divided into three runs (51 trials each run, each condition contains 17 trials). This experiment was performed to explore the neural mechanisms that underlie novelty and usefulness information construction during the creative cognitive process.
Data source location	Institution: Beijing Key Laboratory of Learning and Cognition, School of Psychology, Capital Normal University City: Beijing Country: China
Data accessibility	Repository name: Open Science Framework Direct URL to data: https://osf.io/n9cy7/ DOI: 10.17605/OSF.IO/N9CY7
Related research article	Authors: Jingyuan Ren, Furong Huang, Ying Zhou, Liping Zhuang, Jiahua Xu, Chuanji Gao, Shaozheng Qin, Jing Luo Title: The function of the hippocampus and middle temporal gyrus in forming new associations and concepts during the processing of novelty and usefulness features in creative designs Journal: Neuroimage DOI: 10.1016/j.neuroimage.2020.116751

## Value of the data

• This dataset can benefit creativity researchers to contemplate the critical role of middle temporal gyrus in novelty and usefulness processing of creativity.
• The dataset presented in the article can inspire researchers from other fields such as memory and learning, novel detection.
• The dataset can be used for investigating creativity by combined with other mothed.
• The dataset can be used for decoding mental states during view of familiar objects and novel objects.
• The presented data may guide the future experimental design of creative evaluation processing related to the novelty and usefulness processing of creativity.

## 1 Data description

**Fig. 1:**
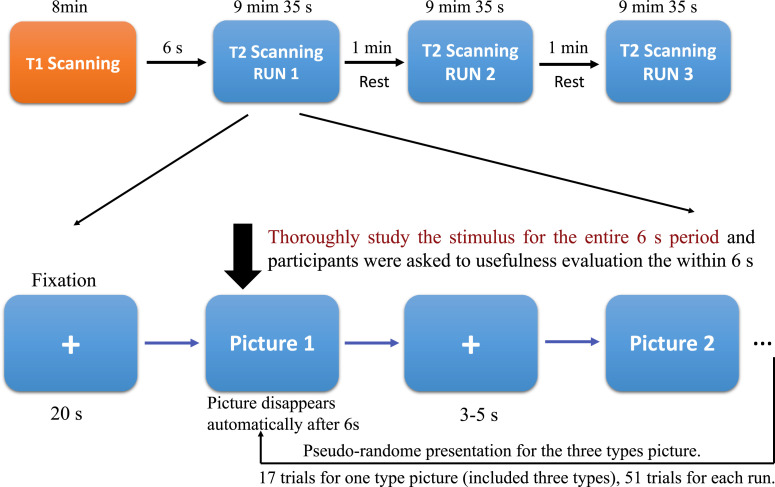
Whole experimental procedure. First, the 3D -T1*-weighted anatomical images were scanned by 8 min, and then scanned T2*-weighted function images for three runs that each run was 9 min 35 s. There was 1 min between the two runs. Each run begins with 20 s “+” fixation. And then, the picture appears for 6 s, followed with 3–5 s jitter. The three types of trials were pseudo-random presented among the three conditions. For the order of the three runs, we also balanced with the different subjects. Participants were asked to comprehend the picture for the entire 6 s and to evaluate the usefulness for each object. There were 51 trials for each run (17 trials for one type).

**Fig. 2:**
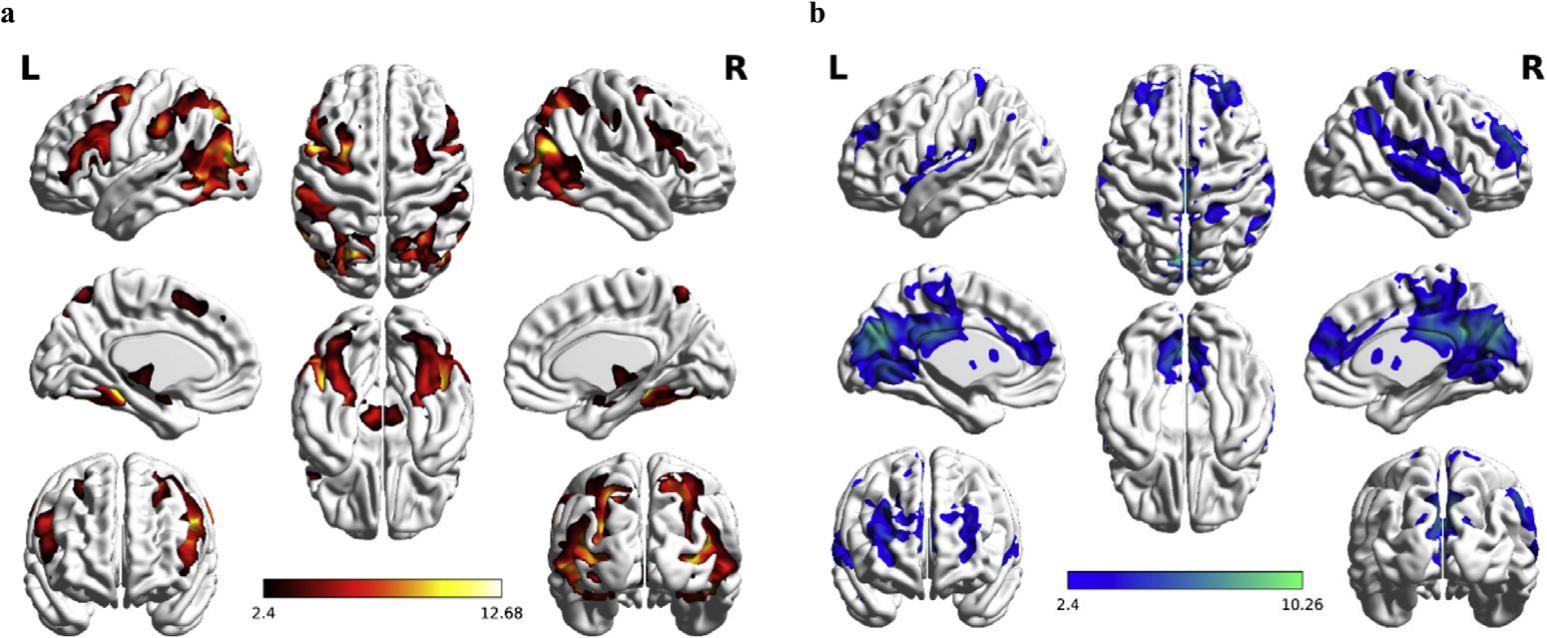
Whole brain analysis of T contrast to separate novelty processing. (a) In the NU-FU comparison, the brain regions involved the medial temporal lobe (MTL), the middle temporal gyrus (MTG), the middle occipital gyrus (MOG), the amygdala, the midbrain (substantia nigra, SN), the thalamus, the fusiform gyrus, the perceptual motion system (pre/postcentral gyrus) and several frontal regions. (b) In the inverse comparison, FU-NU, involved the large brain region of the default mode network (DMN), which includes the precuneus, the cuneus, the medial frontal gyrus and so on (Please see our research paper [6] in Table 2).

**Fig. 3:**
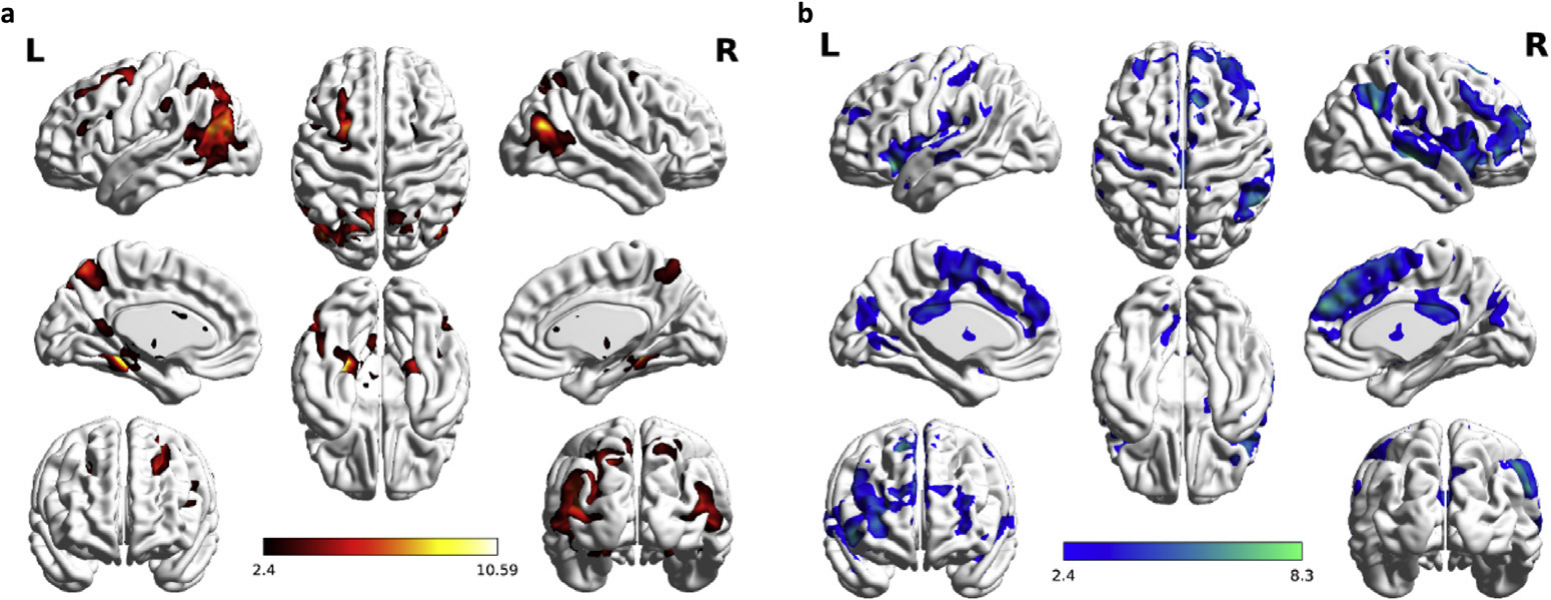
Whole brain analysis of T contrast to separate usefulness processing. (a) In the NU-NS comparison, the brain regions included the medial temporal lobe (MTL), the middle temporal gyrus (MTG), the superior/middle occipital gyrus, the precuneus, and several frontal regions such as superior/middle frontal gyrus. (b) The reverse comparison of NS-NU involved the inferior parietal lobule, the supramarginal gyrus, the insula, the superior temporal gyrus and several frontal regions including the inferior frontal gyrus, the medial frontal gyrus, and the superior frontal gyrus which includes the precuneus, the cuneus, the medial frontal gyrus and so on (Please see our research paper [6] in Table 3).

The novelty and usefulness are two critical features for creativity [1], [2], [3], [4], [5]. The fMRI scans were acquired when participants comprehend the three types of creative design pictures. Each subject's fMRI data, which were divided into three runs. There were 51 trials that averaged contain three conditions (three types of the creative designs) of object picture for each run (17 trials for each condition), and three runs contain 153 trials (51 trials for each condition) (Fig. 1). The released dataset contains the following items: (1) the raw data includes T1-weighted anatomical for each subject, (2) the raw data for fMRI includes three runs for each subject, (3) The basic information of all participants in the MRI scanning (Table 1), (4) the onset time and duration of each trial during the experiment, (5) object stimuli used in the experiment and behavioral post-test, (6) Behavioral post-test data, which was a novelty and usefulness evaluation in a 5-scaled test using the same pictures in fMRI scanning by the same group of participants, and (7) the whole brain activation in novelty processing (Fig. 2) and usefulness processing (Fig. 3), which was supplementary of the original publication in Neuroimage [6].

**Table 1:** The basic information of participants in the scanning.

SubNo	Scanning Time		Gender	Old	Specialty	Note
1	2015/6/4	08:20	Male	23	Traffic Engineering	
2	2015/6/4	09:20	Female	23	–	
3	2015/6/12	08:20	Female	24	Humanities and Social Science	
4	2015/6/12	09:20	Female	25	Management Science and Engineering	
5	2015/6/19	08:20	Male	23	Rural Regional Development	
6	2015/6/25	08:20	Male	24	Mechanical Design and Theory	
7	2015/6/25	09:20	Female	19	Plant Production	
8	2015/7/2	08:20	Female	20	Horticulture Professional	
9	2015/7/2	09:20	Male	21	Seed Science and Technology	
10	2015/7/3	08:20	Female	20	Biological Science	
11	2015/7/3	09:20	Male	23	Electronic Information Science and Technology	
12	2015/7/8	08:20	Male	21	Agricultural Mechanization and its automation	**Deleted**
13	2015/7/8	09:20	Female	20	Industrial Design	
14	2015/7/9	08:20	Female	21	Geographic Information Engineering Science	
15	2015/7/9	09:20	Female	19	Resources and Environment	
16	2015/8/18	08:20	Male	22	Thermal Energy and Power Engineering	**Deleted**
17	2015/8/18	09:20	Male	27	Public Administration	
18	2015/8/19	08:20	Male	23	Computer Science and Technology	
19	2015/8/19	09:20	Male	22	Electrical Engineering and Automation	
20	2015/8/21	08:20	Male	22	Civil Engineering and Water Resources	
21	2015/8/21	09:20	Female	21	Biological Science	

Please Note that SubNo is subject number.

## 2 Experimental design, materials, and methods

### 2.1 Participants

Informed consent was obtained from each subject under a protocol approved by the ethics committee of the Center for Biomedical Imaging Research, Tsinghua University. There were 21 healthy subjects (11 males, mean age ± *s*.d., 22.10 ± 2.05) who were recruited to join the fMRI scanning (19 subjects in formal analysis). Two subjects were excluded in the further analysis, which were the subjects No.12 and No.16 in our dataset. The subject No.12 was reported that he was headache during the scanning, the subject No.16 was had excessive head motion during scanning. For more detail information about subjects, please see the Table 1.

### 2.2 Imaging data acquisition

MR scans were acquired a 3T Philips Achieva 3.0T TX MRI scanner with a 32-channel head coil. A total of 30 slices were acquired every 2 s an echo-planar imaging sequence. T2*-weighted function images: repetition time = 2000 ms, echo time = 35 ms, flip angle = 90°, field of view = 200 mm × 200 mm, 64 × 64 matrix, voxel size = 3.12 mm × 3.12 mm × 4 mm, 30 slices, 4 mm thickness, and no-gap slices. 3D -T1*-weighted anatomical images: 180 slices, repetition time = 7.65 ms, echo time = 3.73 ms, FA = 8°, field of view = 230 mm × 230 mm, voxel size = 1 mm × 1 mm × 1 mm, 1 mm thickness.

### 2.2 Materials and experimental design

The fMRI scanning used 153 pictures that were chosen and modified from the pictures in pretest material designs. Participants were asked to comprehend each design throughout the entire 6 s and respond to the usefulness evaluation for every design via pressing the yes or no button within 6 s. The three types of creativity design were familiar and useful design (FU), novel and useless design (FU), and novel and useful design (NU). Participants performed three runs, with 17 trials per condition each run (each includes three conditions contained 51 trials). For the whole procedure, we first scanned the 3D -T1*-weighted anatomical images, which takes about 8 min, and then scanned T2*-weighted function images for three runs that each run was 9 min 35 s. Between each run had 1 min for subject to rest. Each run begins with 20 s “+” fixation. And then, the picture appears for 6 s, followed with 3–5 s jitter. Each trial displayed 6 s. The three types of trials were pseudo-random presented among the three conditions. Between the two trials was a jitter with 3–5 s. For the order of the three runs, we also balanced with the different subjects (Fig. 1). For more details about the data and analysis methods, please see the original publication in Neuroimage [6].

## Appendix Supplementary materials

Image, application 1
